# Mechanoelectric Response of Single-Crystal Rubrene from Ab Initio Molecular Dynamics

**DOI:** 10.1021/acs.jpclett.1c01385

**Published:** 2021-06-17

**Authors:** Jan Elsner, Samuele Giannini, Jochen Blumberger

**Affiliations:** Department of Physics and Astronomy and Thomas Young Centre, University College London, London WC1E 6BT, United Kingdom

## Abstract

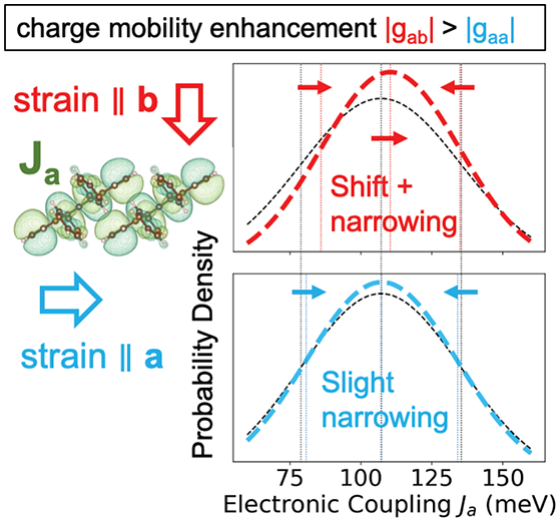

A robust understanding of the mechanoelectric response of organic semiconductors is crucial for the development of materials for flexible electronics. In particular, the prospect of using external mechanical strain to induce a controlled modulation in the charge mobility of the material is appealing. Here we develop an accurate computational protocol for the prediction of the mechanical strain dependence of charge mobility. Ab initio molecular dynamics simulations with a van der Waals density functional are carried out to quantify the off-diagonal electronic disorder in the system as a function of strain by the explicit calculation of the thermal distributions of electronic coupling matrix elements. The approach is applied to a representative molecular organic semiconductor, single-crystal rubrene. We find that charge mobility along the high-mobility direction *a⃗* increases with compressive strain, as one might expect. However, the increase is larger when compressive strain is applied in the perpendicular direction than in the parallel direction with respect to *a⃗*, in agreement with experimental reports. We show that this seemingly counterintuitive result is a consequence of a significantly greater suppression of electronic coupling fluctuations in the range of 50–150 cm^–1^, when strain is applied in the perpendicular direction. Thus our study highlights the importance of considering off-diagonal electron–phonon coupling in understanding the mechanoelectric response of organic semiconducting crystals. The computational approach developed here is well suited for the accurate prediction of strain–charge mobility relations and should provide a useful tool for the emerging field of molecular strain engineering.

One of the major advantages of organic semiconductors (OSs) compared with inorganic materials is their mechanical flexibility.^1^ Whereas flexible displays based on organic light-emitting diodes (OLEDs) are already a multibillion dollar industry,^2^ one can imagine a great deal more in terms of applications—wearable sensors, artificial skin, and soft robotics, to name a few. In some of these applications, one requires robustness of the electronic properties to mechanical strain, for example, in flexible displays, whereas in others, a strong response is desired, for example, in sensors. As such, a detailed theoretical understanding of the mechanoelectric response in organic semiconductors is indispensable and very timely.

A fundamental, molecular-scale understanding of the mechanoelectric response of OS is just about to emerge. To date, the best studied system in this respect is crystalline rubrene^3−7^ (Figure 1), but even for this system, experimental measurements have given rather controversial results. To facilitate the discussion, it is useful to define the mobility–strain enhancement factor, *g*_*ij*_
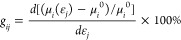
1which measures the fractional change in charge mobility, μ_*i*_, along direction *i* for 1% strain along direction *j*, ε_*j*_. (A negative *g* value indicates that compression, that is, negative strain, leads to an increase in mobility.) Morf et al. found that in tension, the hole mobility of rubrene slightly decreases, as one might expect, *g*_*aa*_ ≃ −4, *g*_*ab*_ ≃ −9,^5^ and similar results were reported by Matta et al., *g*_*aa*_ = −6, *g*_*ab*_ = −21.^6^ It came perhaps as a surprise that the mobility change is larger when strain is applied in the perpendicular direction than in the parallel direction with respect to charge flow (i.e., |*g*_*ab*_| > |*g*_*aa*_|). Most recently, Choi et al. reported radically different results, in terms of both the magnitude and the dependence on the strain direction, *g*_*aa*_ ≃ −70 to −110, *g*_*ab*_ ≃ 0.^7^ In the latter study, particular care was taken to address some of the unaccounted factors in previous experiments. Four-probe measurements were used, taking care of the possible strain dependence of the contact resistance^8^ (whereas previous experiments used a two-probe setup). In addition to the standard field-effect transistor (FET) mobility measurement, the Hall effect was used to obtain the mobility, which has the advantage of probing intrinsic charge carriers.^9^

**Figure 1:**
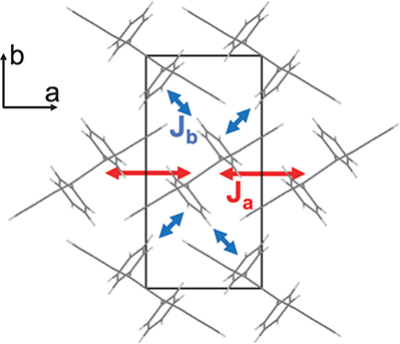
Slice of the conductive *a*–*b* plane of crystalline rubrene. The unit-cell and electronic coupling between molecules in the high mobility direction *a⃗*, *J*_*a*_, and the low mobility direction *b⃗*, *J*_*b*_, are indicated. Electronic coupling in the direction orthogonal to the *a*–*b* plane is negligibly small.

A few theoretical studies on the mechanoelectric properties of rubrene have been carried out as well,^10−12^ broadly agreeing with the experimental studies of Morf et al. and Matta et al. but in contrast with those of Choi et al. Gali et al. used standard band theory, which is known to be problematic for the estimation of charge mobility in molecular organic crystals,^10^ as is small polaron hopping theory.^13^ Ruggiero et al. and Landi et al. used the more suitable transient localization theory (TLT)^14,15^ and treated the off-diagonal electron–phonon coupling and the lattice dynamics in the linear and harmonic approximation, respectively.^11,12,16,17^ Moreover, in the latter study, a semiempirical electronic structure method was used for the lattice dynamics. Because the electronic parameters that govern charge mobilities are very sensitive to fine details of the intermolecular structure and dynamics, the above approximations could tip the balance in favor of one set of experimental results^5,6^ or the other.^7^

Here we address these issues by employing an accurate but computationally more expensive technique for the sampling of intermolecular dynamics governing hole mobility in strained and unstrained rubrene without assuming a harmonic approximation for lattice dynamics or a linear approximation for electron–phonon coupling. Moreover, we employ ab initio molecular dynamics with a van der Waals density functional to sample the room-temperature thermal distribution of electronic couplings between rubrene molecules in the strained and unstrained crystal, and we use them in the framework of TLT to compute charge mobilities. The latter has been shown to give charge mobilities that correlate well with the results of the explicit time propagation of the charge-carrier wave function.^18−21^ Our computations support the experimental results of Matta et al., and they afford a molecular-scale explanation of the peculiar strain dependence of the mobility (|*g*_*ab*_| > |*g*_*aa*_|).

We first address the question of which density functional is most appropriate to describe the structure of unstrained and strained rubrene. To this end, we have investigated a number of van der Waals^22−27^ and dispersion-corrected^28^ density functionals and have compared the optimized lattice constants (at 0 K) to the experimental structure at the lowest temperature reported, 100 K^29^ (Figure 2). Assuming that the remaining effects of the thermal motion and zero point energy are small, the optPBE-vdW functional^22^ appears to best reproduce the experimental structure. Hence, we find that the optPBE-vdW functional gives the best overall performance, and this functional is used in subsequent structural optimizations and ab initio molecular dynamics.

**Figure 2:**
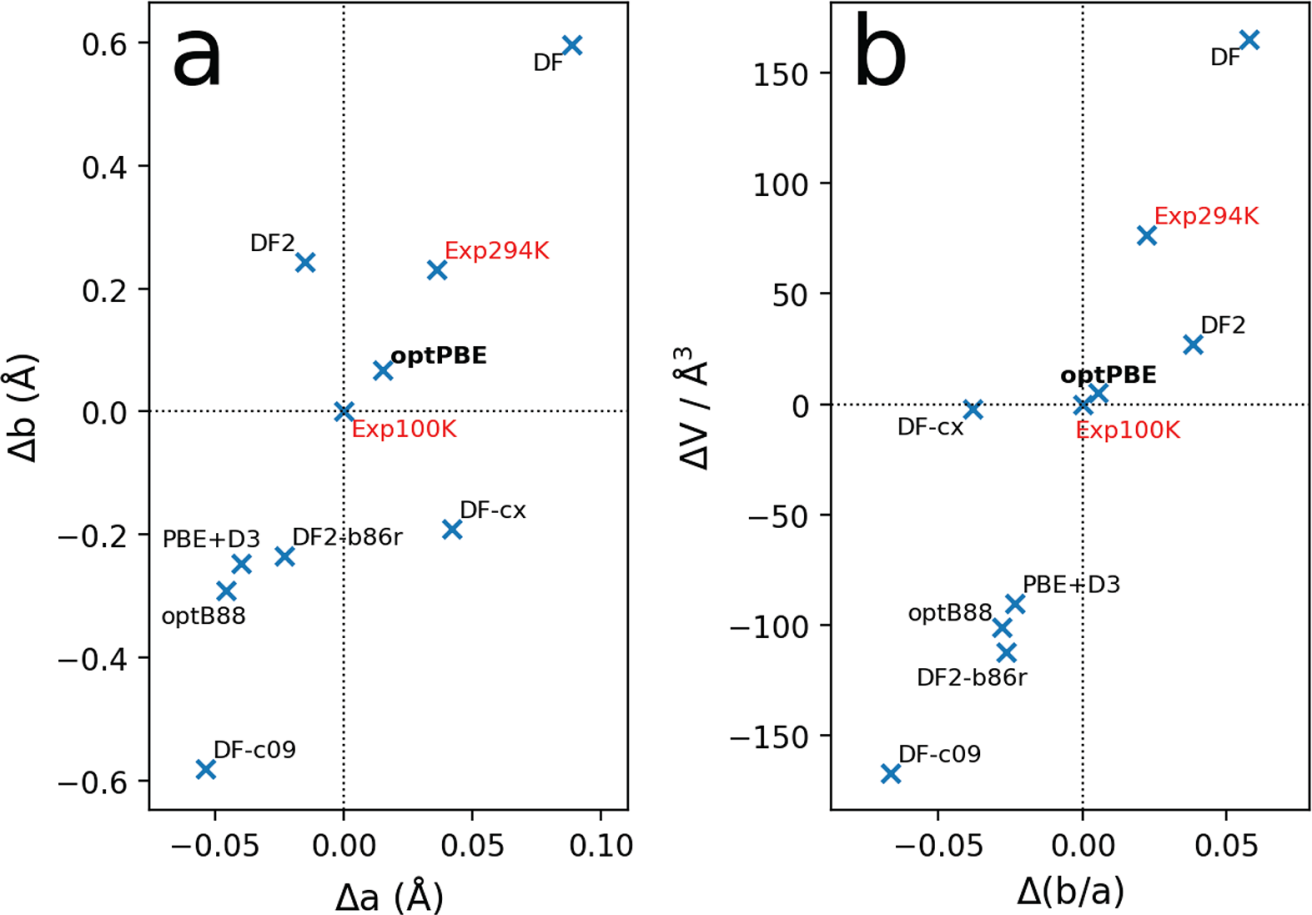
Performance of density functionals in predicting the lattice constants of crystalline rubrene. Deviations of (a) the lattice constants and (b) the volume/anisotropy ratio are shown for several van der Waals and dispersion-corrected density functionals (0 K) with respect to experimental values at low temperature (100 K). The optPBE-vdW functional provides the closest match in both plots.

The structure of unstrained rubrene was obtained by optimizing a 3 × 1 × 1 supercell (12 molecules, 840 atoms) and applying fixed lattice vectors taken from the experimentally determined structure at 294 K.^32^ Finite temperature-related volumetric expansion relative to the optimized 0 K structure and zero-point motion effects on the cell dimensions are thus included in an empirical manner. Strained structures along *a⃗* and *b⃗* were obtained by scaling the relevant lattice parameter followed by reoptimization of all atomic positions at fixed strained lattice parameters. Electronic couplings were calculated for dimers taken from the unstrained and strained structures using the projector-operator-based diabatization (POD) technique^30^ in combination with the Perdew–Burke–Ernzerhof (PBE) density functional and scaling of the resultant values by a uniform constant of 1.325. This method is referred to as sPOD/PBE. The scaling factor was obtained from a best fit to ab initio reference values for the HAB11 database of organic dimers,^31^ resulting in a very small mean relative error of only 3.5%. See the Supporting Information (SI) for further details.

The strain dependence of electronic coupling is shown in Figure 3. We focus on the electronic coupling along the high-mobility direction *a⃗*, *J*_*a*_ (Figure 3a), and note that similar results apply to coupling along the *b⃗* direction, *J*_*b*_ (Figure 3b). We go to larger values of strain in the case of compression (negative strain) because we are primarily interested in exploring how far we can increase the mobility. Strain values of up to ∼1% and compression along both directions *a⃗* (blue) and *b⃗* (orange) result in a linear increase in *J*_*a*_. We notice that the coupling increases slightly more strongly when strain is applied along *b⃗*, in line with previous studies.^6,10,11^ At larger strain values, this difference becomes very pronounced. Whereas *J*_*a*_ keeps increasing for strains along *b⃗* > ∼6%, it saturates to a plateau value for strains along *a⃗* > ∼6%. We note that in the experiment, rubrene samples under strains of >∼0.4% are prone to forming cracks and become unstable on macroscopic time scales; however, from a theoretical perspective, it is of interest to explore the response at higher strain values because the material might sustain these when applied for very short durations of time.

**Figure 3:**
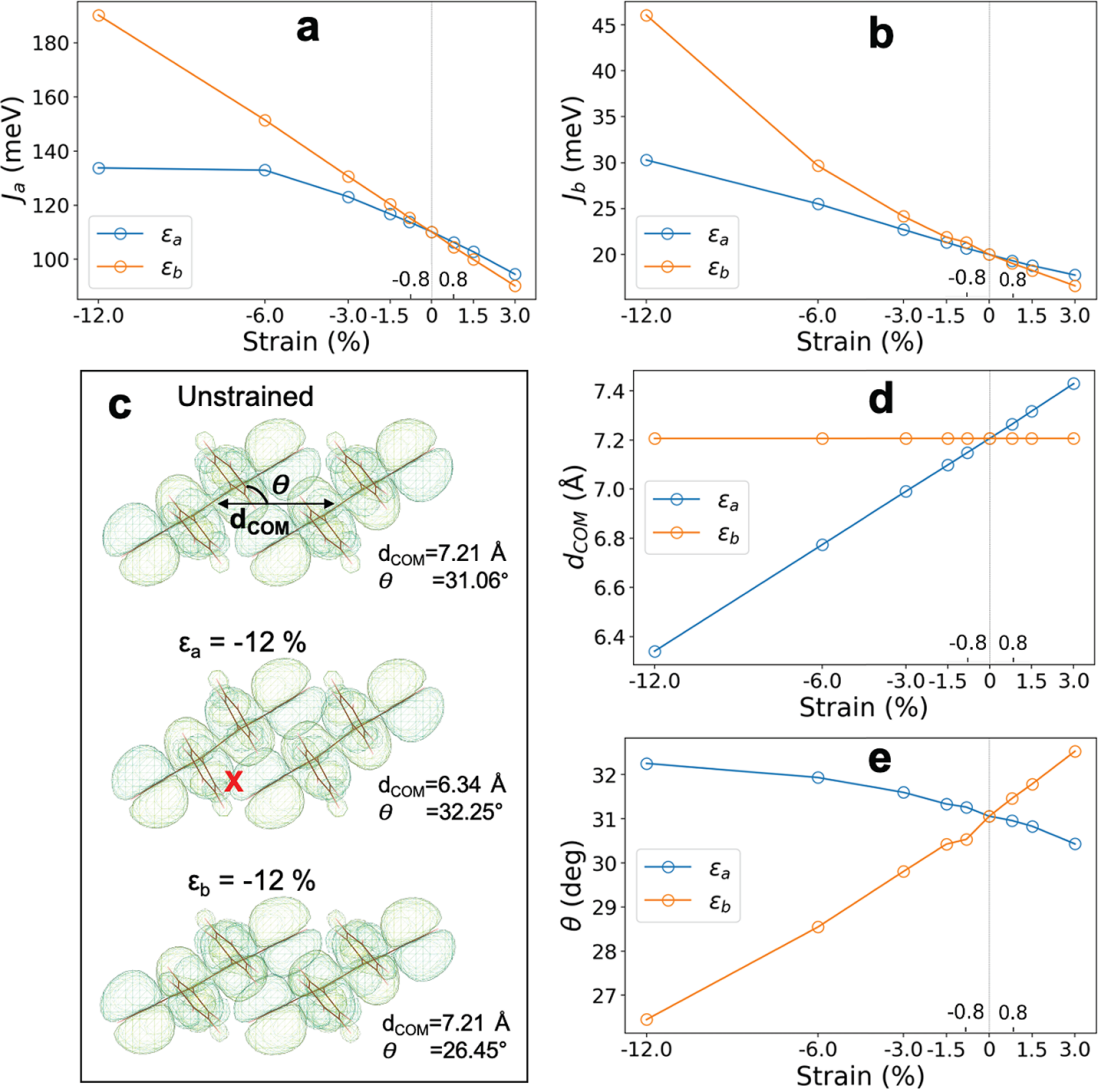
Strain dependence of electronic coupling in rubrene. (a) *J*_*a*_ and (b) *J*_*b*_ at minimum energy geometry for strains between −12 and +3% along *a⃗* (blue) and *b⃗* (orange). (c) Visualization of the superimposed highest occupied molecular orbitals used for the calculation of *J*_*a*_, as obtained from the POD method, for the unstrained case and −12% strain along *a⃗* and *b⃗*. We mark the distance between the center of mass of each molecule (*d*_COM_) and the tilt angle θ. For −12% strain along *a⃗*, the red “X” marks the onset of overlap between two lobes that contributes destructive interference, resulting in the observed plateau in *J*_*a*_ versus ε_*a*_ (panel a). (d) Variation in *d*_COM_ and (e) θ as a function of strain along *a⃗* (blue) and *b⃗* (orange).

The dependence of the coupling enhancement on the strain direction can be rationalized by analyzing the structural response to strain in terms of the center of mass distance, *d*_COM_, and the tilt angle, θ, of the rubrene molecules and their effect on the overlap between the highest occupied molecular orbitals (HOMO) that mediates the coupling (Figure 3c). Strain along *a⃗* leads to a linear decrease in *d*_COM_ (Figure 3d), whereas the tilt angle of the molecules, θ, increases only slightly (Figure 3e). The decrease in *d*_COM_ results in the onset of destructive interference between orbital lobes (point marked with “X” in Figure 3c), counterbalancing any increase in overlap arising elsewhere. The situation is markedly different for strain along *b⃗*: The tilt angle decreases strongly, whereas *d*_COM_ remains virtually unchanged. Destructive interference of the kind seen above does not occur. On the contrary, the “θ-twist” motion ensures progressively constructive interference and increasing orbital overlap as θ decreases, resulting in progressively increasing electronic coupling.

Whereas the electronic couplings calculated above for 0 K give first clues with regard to the effect of mechanical strain, it is the thermal distribution of the couplings that ultimately governs the charge mobility in OS. We used DFT(optPBE)-MD to sample the thermal motion of the rubrene crystal at zero strain, −0.8% strain along *a⃗*, and −0.8% strain along *b⃗* at room temperature. For each type of electronic coupling, *J*_*a*_ and *J*_*b*_, we considered two independent dimers in the supercell; that is, we calculated four electronic coupling time series, *J*_*a*,1_(*t*), *J*_*a*,2_(*t*), *J*_*b*,1_(*t*), and *J*_*b*,2_(*t*), for each value of strain. Two of these time series, *J*_*a*,1_(*t*) and *J*_*b*,1_(*t*), are shown in Figure 4 for each value of strain, and numerical results are summarized in Table 1.

**Figure 4:**
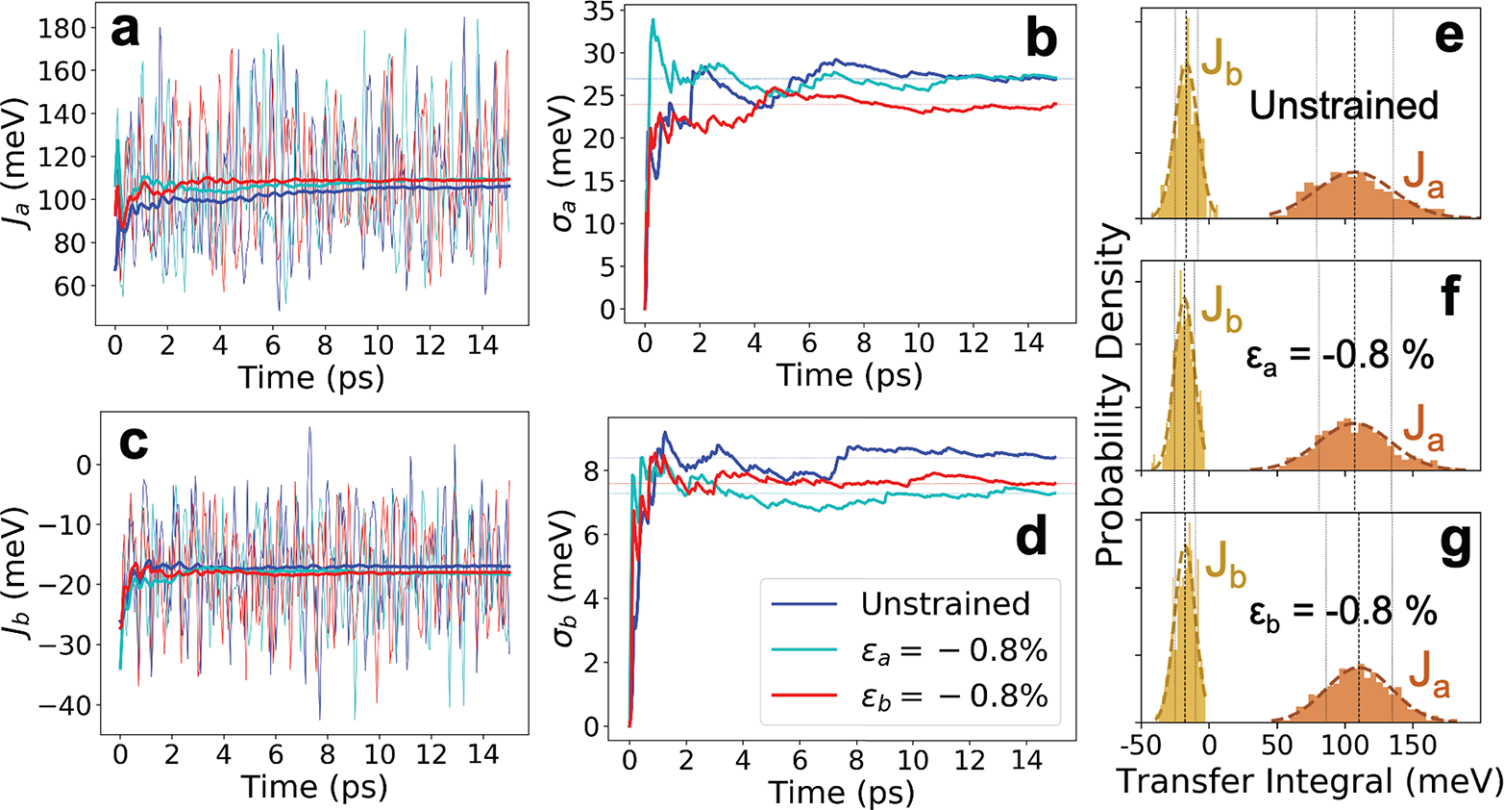
Thermal fluctuations of electronic coupling in unstrained and strained rubrene at 290 K. Time series for (a) *J*_*a*_ and (c) *J*_*b*_ with the accumulated average in bold. The unstrained trajectory is shown in dark blue, ε_*a*_ = −0.8% is in cyan, and ε_*b*_ = −0.8% is in red. Accumulated root-mean-square fluctuations for (b) *J*_*a*_, σ_*a*_ and (d) *J*_*b*_, σ_*b*_. (e–g) Histograms of the final distributions of *J*_*a*_ and *J*_*b*_. Gaussian fits are shown with dashed lines. The data in panels a–d are for the electronic coupling time series of a single *J*_*a*_ and *J*_*b*_ dimer (*J*_*a*,1_, *J*_*b*,1_), whereas the overall distributions, panels e–g, include all calculated electronic couplings (*J*_*a*,1_, *J*_*a*,2_, *J*_*b*,1_, *J*_*b*,2_). A significant decrease in σ_*a*_ (panel b) and a corresponding narrowing of the *J*_*a*_ distribution (panel g) can be seen for ε_*b*_ = −0.8%.

**Table 1:** Dependence of Electronic Couplings for Hole Transfer in Rubrene, *J*_*a*_ and *J*_*b*_, on Mechanical Strain, ε^a^

ε	*J*_*a*_	⟨*J*_*a*_⟩	σ_*a*_	*J*_*b*_	⟨*J*_*b*_⟩	σ_*b*_	τ_TLT_
0 (unstrained)	110.2^b^	107.1 ± 0.9^c^	28.2 ± 1.3^c^	–20.0^b^	–17.0 ± 0.1^c^	7.7 ± 0.8^c^	0.068^d^
–0.8% along *a⃗*	113.8^b^	107.3 ± 2.1^c^	26.6 ± 0.6^c^	–20.7^b^	–17.7 ± 0.7^c^	7.9 ± 0.6^c^	0.066^d^
–0.8% along *b⃗*	115.3^b^	110.3 ± 0.9^c^	24.4 ± 0.4^c^	–21.3^b^	–17.9 ± 0.1^c^	7.2 ± 0.4^c^	0.066^d^
0, ref ^11^	112.4		34.2	–25.9		10.3	0.079
0, ref ^12^	149.2		37.8	–20.3		9.2	0.159

a All values are in millielectronvolts unless otherwise indicated.

b sPOD/PBE electronic coupling for structures obtained by geometry optimization at fixed lattice dimensions using the optPBE-vdW functional. The initial structure and lattice dimensions of unstrained rubrene were taken from the experiment at 294 K.^32^

c Thermal average or root-mean-square fluctuations of sPOD/PBE electronic couplings at 290 K, obtained from DFT-MD trajectories generated with the optPBE-vdW density functional. The error bars are equal to half the difference between the values obtained from one set of dimers (*J*_α,1_; α = *a*, *b*) and the other (*J*_α,2_; α = *a*, *b*).

d Time constant (in ps(rad)^−1^) corresponding to the harmonic mean of the vibrational frequencies, weighted by the average power spectrum of *J*_*a*_ electronic coupling fluctuations.

Importantly, we observe a significantly larger decrease in the root-mean-square fluctuations of the couplings, σ_*a*_ = ⟨(*J*_*a*_ – ⟨*J*_*a*_⟩)^2^⟩^1/2^, corresponding to a decrease in off-diagonal disorder, for compression along *b⃗* (13% decrease) compared with compression along *a⃗* (6% decrease); see Table 1. To gain further insight into the modes responsible for the dynamics, we calculate the spectral density function of the electronic coupling time series from the cosine transformation of the autocorrelation function. The running integral of the spectral density yields the cumulative disorder, including all frequencies up to ω, σ_α_(ω), allowing us to quantify the relative contribution of each mode
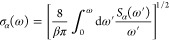
2where *S*_α_(ω) is the spectral density for time series *J*_α_ (α = *a*, *b*) and β = (*k*_B_*T*)^−1^. (See the SI for further details.) Including all frequencies ω → ∞ returns the root-mean-square fluctuation of the time series. Figure 5 displays plots of the spectral densities and corresponding σ(ω) functions for *J*_*a*_ (Figure 5a) and *J*_*b*_ (Figure 5b) over all values of strain, using the same *J*_*a*/*b*_ time series considered in Figure 4a–d. We observe that in the case of strain along *b⃗*, there is a noticeable reduction in the spectral density amplitude for *J*_*a*_ in the frequency range 50–150 cm^–1^, ultimately leading to a smaller overall root-mean-square fluctuation, as evidenced in Table 1.

**Figure 5:**
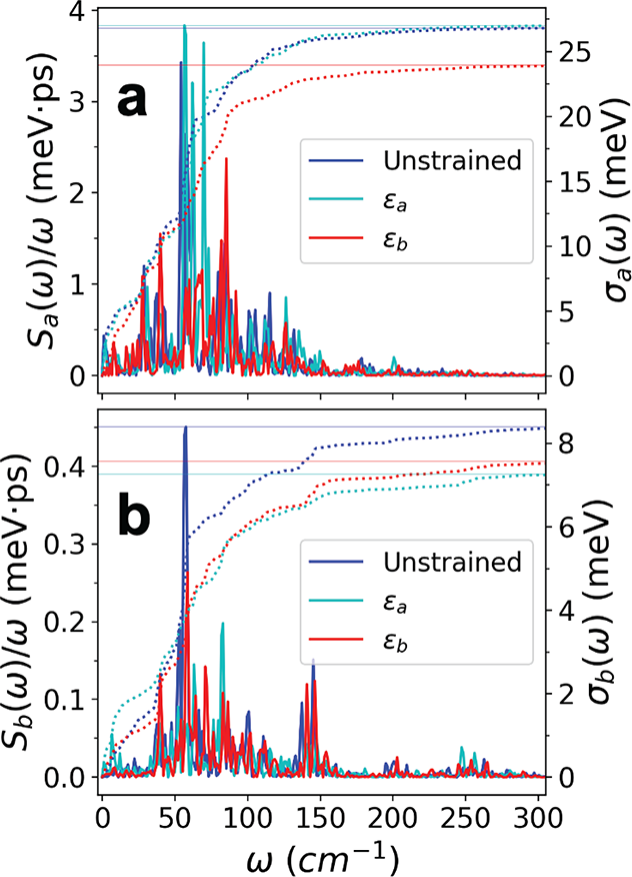
Spectral density functions and cumulative disorder, σ(ω) (eq 2), for (a) the *J*_*a*_ time series and (b) the *J*_*b*_ time series corresponding to the same dimers used in Figure 4a–d (*J*_*a*,1_(*t*), *J*_*b*,1_(*t*)). In panel a, we observe that σ_*a*_ is smaller for strain along *b⃗* due to a suppression of the spectral density amplitude in the frequency range 50–150 cm^–1^.

Using the DFT-MD electronic coupling distributions, we calculate the hole mobility for unstrained and strained rubrene using TLT.^14,15^ Three different flavors of TLT are considered to account for the site energy (or diagonal energy) fluctuations. In method 1, all diagonal elements of TLT Hamiltonian are set to zero. In method 2, site energy fluctuations due to intramolecular modes are included by sampling from a Gaussian distribution with width , where λ = 0.152 eV is the inner-sphere reorganization energy. In method 3, the electronic coupling distributions are renormalized with an effective, λ-dependent band renormalization factor.^33,34^ See the SI for further details and Table S1 for a summary of numerical values. Agreement with the currently accepted experimental mobility of unstrained, highly pure single-crystalline rubrene, ∼15 cm^2^ V^–1^ s^–1^,^35^ is reasonable, within a factor of 3 for all three methods.

The mobility–strain enhancement factors, as defined in eq 1, are summarized in Table 2. For all three methods, we obtain *g*_*aa*_, *g*_*ab*_ < 0, and |*g*_*aa*_| < |*g*_*ab*_|. Importantly, the values are in good agreement with the experimental measurements of ref (6), with deviations of *g* values of less than a factor of 2. Our key result, |*g*_*aa*_| < |*g*_*ab*_|, is due to the significant suppression of electronic coupling fluctuations (off-diagonal electron–phonon coupling) in the range of 50 to 150 cm^–1^ as well as a small increase in the mean electronic couplings when strain is applied along *b⃗*. (See the discussion above and Table 1.) The suppression of *J*_*a*_ coupling fluctuations is not as effective when strain is applied along *a⃗* (see Figure 5a), and we see only a negligible increase in the mean electronic couplings, which explains the observed sensitivity of the mobility enhancement on the strain direction.

**Table 2:** Strain–Mobility Enhancement Factors Calculated from Equation 1 Using the Transient Localization Theory (TLT) and Results from Other Computational (comp) and Experimental (exp) Studies^a^

		*g*_*aa*_	*g*_*ab*_	*g*_*ba*_	*g*_*bb*_
this work^b^	comp	–12 ± 2	–41 ± 8	–20 ± 2	–43 ± 7
this work^c^	comp	–8 ± 8	–25 ± 1	–17 ± 10	–29 ± 1
this work^d^	comp	–9 ± 1	–32 ± 7	–17 ± 5	–33 ± 5
ref ^5^	exp	–4	–9		
ref ^6^	exp	–6	–21		
ref ^7^	exp	–70 to −110	0		
ref ^10^	comp	–9	–12	–15	–18
ref ^11^	comp	+4	–12	+4	–13
ref ^12^	comp	–8	–16	–10	–16

a *g* values presented are obtained by using the overall distributions of electronic couplings (see Table 1) as input to TLT calculations. The error bars are equal to half the difference between the *g* values calculated using the electronic coupling distributions of one set of dimers (*J*_*a*,1_, *J*_*b*,1_) and the other (*J*_*a*,2_, *J*_*b*,2_).

b No diagonal electron–phonon coupling; that is, all site energies are set to zero.

c Site energy fluctuations from the Gaussian distribution corresponding to λ = 0.152 eV.

d Diagonal electron–phonon coupling accounted for via band renormalization.

The two previous computational studies of Landi et al. and Ruggiero et al. arrived at similar results, in particular, that |*g*_*aa*_| < |*g*_*ab*_|. However, Ruggerio et al. did not find a similar suppression of electronic coupling fluctuations with compressive strain, and, surprisingly, *g*_*aa*_ was reported to be slightly positive. Their calculations differed in a number of important aspects from our calculations; in particular, they used the linear electron–phonon and harmonic approximations (not used here), and there are differences with regard to the unit-cell dimensions (DFT minimum vs experiment), the method for the electronic coupling calculations, and the DFT functional used, which can all contribute to the differences observed. Here we investigated the accuracy of the linear approximation for electron–phonon coupling in some detail; see the SI. Our calculations indicate that this approximation can give errors in mean electronic couplings that are on the same order of magnitude as the (small) effect of 0.8% compressive strain. Hence, this approximation can become problematic in situations where high accuracy is required like in the present application. Unfortunately, a consistent investigation of the accuracy of the harmonic approximation could not be carried out because phonon calculations for supercells as large as the ones used in the current ab initio MD simulations remain impractical.

According to our calculations and the experiments of refs (5) and (6), the mobility enhancement at strain values that can be realized in the experiment without causing plastic deformation (ε < 0.4%) is rather modest (<17%). From a theoretical perspective, it is of interest to explore what strain values would be required to achieve more significant mobility enhancements. Because the simple linear relation eq 1 is no longer valid for large compressions, we take the computed electronic coupling values displayed in Figure 3 for −12% compression and the root-mean-square fluctuations obtained from DFT-MD at −0.8% compression. Because we expect that electronic coupling fluctuations will further decrease with increasing compression, this is most likely an underestimate, and we therefore consider the values obtained as lower limits. We obtain μ_*a*_ > 120 cm^2^ V^–1^ s^–1^ and μ_*a*_ > 48 cm^2^ V^–1^ s^–1^, corresponding to a 3.5-fold and 1.5-fold mobility enhancement for strain along *b⃗* and *a⃗*, respectively (band-renormalized, TLT method 3). These extremely high mobility values are unfortunately not experimentally accessible because structures under such high compressions will quickly undergo mechanical failure.

In summary, we have used DFT-MD simulations with the optPBE-vdW functional and TLT to calculate the strain dependence of mobility in single-crystalline rubrene. Our results are in good agreement with the experimental studies of refs (5) and (6). In particular, they explain the somewhat counterintuitive observation that the mobility enhancement in rubrene is larger when strain is applied in the perpendicular direction than in the parallel direction with respect to the electron flow. However, our study (and the one of Landi et al.) disagrees with the most recent and very carefully conducted experimental work of Choi et al. It is possible that interfacial sample/substrate effects particular to that specific experiment or remaining structural defects, not accounted for in the computational models, are responsible for the discrepancy. However, further experimental studies will be necessary to give a more conclusive answer.

The intrinsic mechanoelectric response in pure, single-crystalline rubrene is found to be relatively modest, which is an advantage for flexible electronics applications where the electronic properties should remain robust with respect to mechanical strain. However, this also means that applying gentle external compressive strain as a means to boost the charge mobility is not very effective for this material. Yet, recent theoretical evidence suggests that the mechanoelectric response may strongly depend on the organic molecule under consideration,^12^ more specifically, on the nodal shape and the relative orientation of the charge-mediating molecular frontier orbitals in the crystal. Hence it might be possible in the future to develop organic semiconducting materials that show either weak or strong dependence on the external strain, as desired for a given electronic application.

## Supporting Information Available

Details of the electronic structure calculations, structural optimizations, molecular dynamics settings, electronic coupling calculations using the POD method, assessment of the linear approximation for non-local electron-phonon coupling, equations defining the cumulative disorder, overview of TLT and discussion of the different flavors used to account for local electron–phonon contributions, and a table listing numerical values for mobilities obtained from TLT (PDF)
