# Supplementary material for: Mechanoelectric Response of Single-Crystal Rubrene from Ab Initio Molecular Dynamics
Source: J Phys Chem Lett. 2021 Jun 17;12(25):5857–63. doi: 10.1021/acs.jpclett.1c01385 (PMC8256417; doi:10.1021/acs.jpclett.1c01385)
Supplement: Supplementary file 1 — jz1c01385_si_001.pdf [file jz1c01385_si_001.pdf]

Mechanoelectric Response of Single Crystal Rubrene from Ab-initio Molecular Dynamics

Jan Elsner, Samuele Giannini, and Jochen Blumberger*

Department of Physics and Astronomy and Thomas Young Centre, University College London, London WC1E 6BT, UK.

E-mail: j.blumberger@ucl.ac.uk

Supporting Information

Structural Optimisations

All electronic structure calculations were carried out in the CP2K package.[1–6] The optPBE-vdW density functional of Klimes *et al.*[7,8] was used along with a DZVP basis set[9] and GTH pseudopotentials.[10] All optimizations utilized the BFGS algorithm.[11] Variable-cell optimizations were carried out on the unit cell using a $4 \times 2 \times 1$ Monkhorst-Pack reciprocal space grid.[12] The maximum force, displacement and pressure thresholds for the optimization were set to 5.0×10^{-5} Ha/Bohr, 1.0×10^{-4} Bohr and 5.0 bar, respectively. A plane wave cutoff of 1150 Ry was used for representing the density and a relative cutoff of 80 Ry for real-space integration using a multi-grid method. Geometry optimisations with fixed lattice parameters were carried out using a $3 \times 1 \times 1$ supercell (12 molecules, 840 atoms) at the Gamma point. We note that the trend in electronic coupling with strain was found to be well converged with respect to a $4 \times 2 \times 1$ supercell, validating our use of a $3 \times 1 \times 1$ supercell at the Gamma point. The efficient orbital transformation method[13] was used for the SCF calculations and the third order ASPC algorithm was used for wavefunction extrapolation.[14] Cell vectors for the unstrained structure were fixed to the experimental values at 294 K; $a = 7.206$ Å, $b = 14.442$ Å and $c = 26.965$ Å.[15] Strained structures were obtained by scaling the relevant lattice vector and re-optimising the structure with the above-mentioned settings. The maximum force and displacement thresholds were set to 7.5×10^{-5} Ha/Bohr and 5.0×10^{-4} Bohr, respectively. The plane wave cutoff and relative cutoff were set to 600 Ry and 50 Ry, respectively.

Calculation of electronic coupling matrix elements

Electronic couplings were calculated using the projection operator-based diabatization (POD) method.[16,17] In this scheme, the self-consistent DFT Kohn-Sham Hamiltonian of the dimer in question is represented in an orthogonalised atomic orbital basis and is partitioned into donor and acceptor blocks. Each block is separately diagonalised, defining unitary trans-

formations which are used to transform the off-diagonal blocks. The resulting off-diagonal matrix elements may be identified as electronic couplings between the block-diagonal donor and acceptor states. The Kohn-Sham DFT calculations were carried out with the PBE functional, a DZVP basis set and GTH pseudopotentials on the neutral dimer. After block diagonalization, the Hamiltonian matrix element between the HOMO POD states on each molecule is taken as the electronic coupling matrix element.

DFT-MD

DFT-MD was carried out for the unstrained structure and structures at 0.8 % compression along both $\vec{a}$ and $\vec{b}$. The optPBE-vdW density functional was used, along with a DZVP basis set, GTH pseudopotentials and a $3 \times 1 \times 1$ supercell. Cell vectors for the unstrained structure were fixed to the experimental values at 294 K[15] and strained structures were obtained by scaling the relevant lattice vector and re-optimising the structure. The time step was set to 0.5 fs. The plane-wave cutoff and relative cutoff were set to 300 Ry and 30 Ry, respectively. We equilibrated the systems for 2 ps in the NVT ensemble at 290 K using the Canonical Sampling through Velocity Rescaling algorthim[18] with a progressively increasing time constant, followed by 15 ps in the NVE ensemble. In all cases, the total energy was conserved to less than 4×10^{-7} Ha$(\text{atom})^{-1}(\text{ps})^{-1}$, validating the accuracy of our electronic structure calculations. POD couplings were calculated from snapshots at intervals of 50 fs along the NVE trajectory using two independent dimers in the supercell for J_a ($J_{a,1}$, $J_{a,2}$) and for J_b ($J_{b,1}$, $J_{b,2}$), leading to a total of 600 J_a and 600 J_b values along each trajectory.

Linear Approximation for Electronic Couplings

Here we test the approximation that the electronic coupling has a linear dependence on phonon coordinate, as assumed by Ruggiero *et al.*[19] and Landi *et al.*[20] Within this approximation we have:

$$J(\vec{Q}) = J_0 + \vec{\nabla} J \cdot \vec{Q} \quad \text{(S1)}$$

where J is the electronic coupling, J_0 is the electronic coupling at minimum energy geometry and $\vec{Q}$ is a vector of Cartesian displacements from equilibrium. $\vec{\nabla} J$ can be calculated through finite differences by successively displacing the coordinates of each atom in the dimer starting from the equilibrium geometry and taking the difference between the sPOD electronic coupling at positive and negative displacements along each direction. We used an increment of 0.01 Å for the finite differences, and we verified that values in the range 0.002 to 0.02 Å gave very similar results. In Fig. S1 we show the correlation between electronic couplings J_a calculated using the full sPOD calculation vs the linear approximation Eqn. S1.

The overall distributions we obtain from the full sPOD calculation and the linear approximation are (106.2 ± 26.9) meV and (111.4 ± 26.6) meV, respectively. We see that the linear approximation yields a slightly larger mean value and a very similar RMS fluctuation. We note that the difference in these mean values is actually larger than the differences observed between strained and unstrained structures, hence while this approximation may be very good for studying charge transport in most cases, it is perhaps not appropriate for resolving the very small changes resulting from straining a sample. Due to the large number of sPOD calculations required for the calculation of $\vec{\nabla} J$ (840 calculations in total), we have only calculated this quantity for J_a in the unstrained state, therefore we cannot assess how well this approximation reproduces the trend in mobility with strain, as defined by the g values (Eqn. 1 of the main text).

Frequency Analysis

Denoting the autocorrelation function of the time series $J_a(t)$ as $c_a(t)$, we have

$$c_a(t) = \langle \delta J_a(0) \delta J_a(t) \rangle \quad \text{(S2)}$$

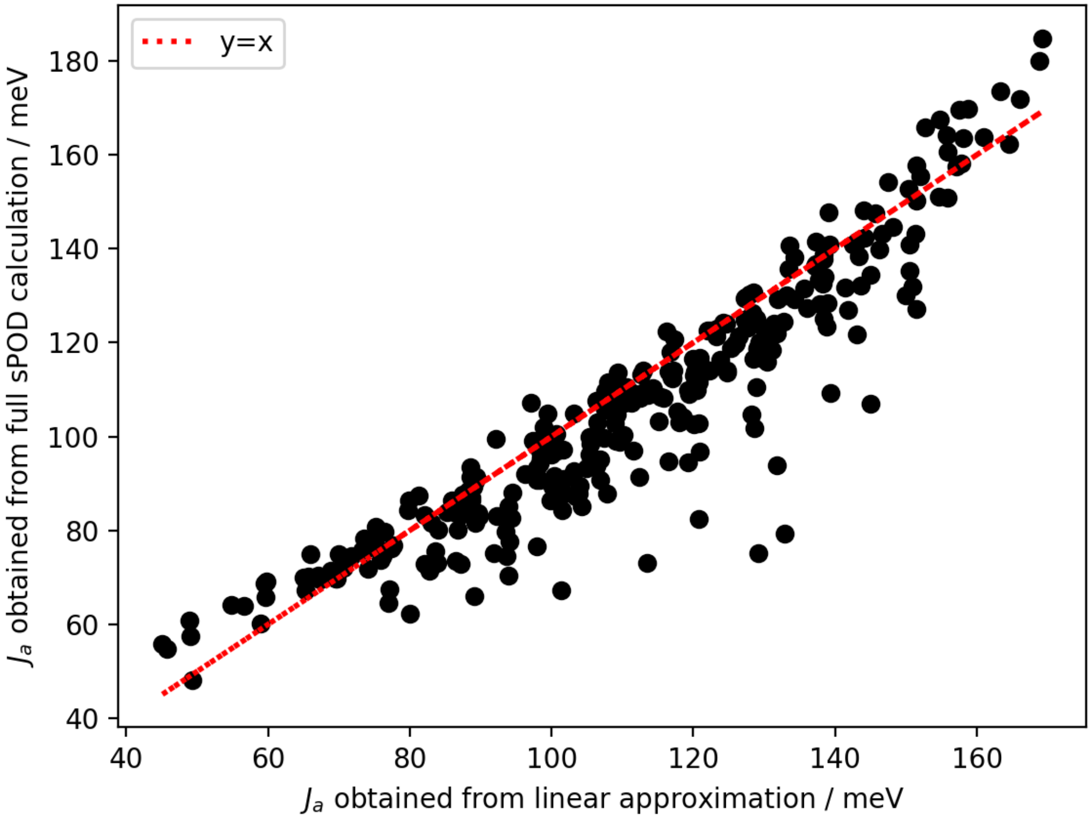

Figure S1: Correlation between electronic couplings J_a calculated using full sPOD calculation vs the linear approximation, Eqn. S1. We observe a relatively good correlation, with some spread. There is a slight tendency for the linear approximation to overestimate the electronic coupling, particularly in the mid-coupling region.

where $\delta J_a(t) = J_a(t) - \langle J_a \rangle$. The spectral density function, $S_a(\omega)$, is obtained from the cosine transform of $c_a(t)$ (see, e.g., Ref. 21),

$$\frac{S_a(\omega)}{\omega} = \frac{\beta}{4} \int_0^\infty dt c_a(t) \cos \omega t, \tag{S3}$$

where $\beta = 1/k_B T$, allowing $c_a(t)$ to be expressed in terms of the inverse transformation:

$$c_a(t) = \frac{8}{\beta \pi} \int_0^\infty d\omega \, \frac{S_a(\omega)}{\omega} \cos \omega t. \tag{S4}$$

Recognising that the autocorrelation function at $t = 0$, $c_a(0) = \langle \delta J_a^2 \rangle$, is equivalent to

the variance of J_a, we can calculate the running integral of Eqn. S4 with $t = 0$ up to a frequency ω, which corresponds to the variance of the time series including all frequency contributions up to ω. Working in terms of the root-mean-square fluctuation $\sigma_a(\omega) = \sqrt{c_a(t=0)}$, we have:

$$\sigma_a(\omega) = \left[\frac{8}{\beta\pi}\int_0^{\omega} d\omega' \; \frac{S_a(\omega')}{\omega'}\right]^{\frac{1}{2}}, \tag{S5}$$

which is Eqn. 2 of the main text.

Transient Localisation Theory

Transient Localization Theory (TLT) starts from the assumption of large amplitude fluctuations and, using the Kubo-Greenwood formalism in the relaxation time approximation, derives the following expression for the charge mobility[22,23]

$$\mu_{x(y)} = \frac{e}{k_B T}\frac{\bar{L}^2_{x(y)}}{2\tau} \tag{S6}$$

where e is the elementary charge, k_B is the Boltzmann constant, T is temperature, τ is the timescale of lattice vibrations, L is the so-called transient localization length and $\bar{L}$ denotes the average over multiple realisations of disorder. The squared transient localization length is given by

$$L^2_{x(y)}(\tau) = \frac{1}{Z}\sum_{n,m} e^{\beta E_n}|\langle n|\hat{j}_{x(y)}|m\rangle|^2 \frac{2}{(\hbar/\tau)^2 + (E_m - E_n)^2} \tag{S7}$$

where Z is the partition function, $\hat{j}$ is the current operator and ($|n\rangle$, E_n) refer to the eigenstates and eigenvalues of a Hamiltonian with a particular realisation of disorder. In practice, disordered Hamiltonians are constructed by sampling from a specified Gaussian distribution of electronic couplings (and possibly also a distribution of site energies) for a given supercell size. The average localization length, $\bar{L}$ is obtained by averaging over the tran-

sient localization lengths corresponding to all Hamiltonians considered. Finally, mobility is calculated using Eqn. S6. TLT mobility calculations for each value of strain were carried out using freely available code[24] (https://github.com/CiuK1469/TransLoc, code version 0.4, downloaded 14th December 2020). We used a 39×26 supercell under periodic boundary conditions, which gave well-converged results in all cases, and we averaged over the transient localization lengths obtained from 100 distinct disordered Hamiltonians. The time constant τ was obtained from the harmonic mean of the vibrational frequencies, weighted by the power spectrum for J_a, $S_a(\omega)/\omega$, as defined in Eqn. S3. Since we calculated the J_a time series for two distinct dimers, $J_{a,1}(t)$ and $J_{a,2}(t)$, the average of these two spectra was used. Taking the harmonic mean of the vibrational frequencies is preferable to using, for example, the frequency corresponding to the peak of $S_a(\omega)/\omega$, since many modes contribute to σ_a, as evident in Fig. 5 of the main text. We note that in practice the TLT mobility is only very weakly dependent on the choice of τ,[25] and using the peak of $S_a(\omega)/\omega$ instead yields very similar values.

Three approaches were considered. First, we simply provided our DFT-MD electronic coupling distributions as input, setting all diagonal elements of our TLT Hamiltonians to zero. Second, we included site energy fluctuations with $\sigma_E = \sqrt{2k_BT\lambda} = 88.65$ meV, which is the expected value for a system where a single molecule is charged throughout the dynamics. Here, λ is the reorganisation energy which we have previously calculated to be 0.152 eV.[26] This value for σ_E is very similar to the value obtained from a 15 ps (uncharged) classical MD trajectory (General Amber Force Field[27] with a $34 \times 2 \times 2$ supercell), $\sigma_E = 88.05$ meV, where the magnitude of site energy fluctuations is obtained from the standard deviation of the classical force field energy of snapshot configurations with a single molecule in the charged state and all other molecules neutral. We note that site energy fluctuations are primarily due to fast intramolecular vibrations which should ideally be quantized, therefore the results obtained from this approach should be considered with caution. Finally, we accounted for local electron-phonon coupling contributions through a renormalization of electronic coupling

distributions using the scaling parameter[28,29]

$$\alpha = \exp[-\lambda \coth\left(\hbar\omega_H/2k_BT\right)/2\hbar\omega_H] \tag{S8}$$

where λ is the reorganization energy and ω_H is the effective frequency of intramolecular vibrations, here set to $\omega_H = 1200$ cm^{-1} as suggested in previous studies.[28,30] At $T = 290$ K this evaluates to $\alpha = 0.60$. In this case, all site energies were set to zero, since the local electron-phonon coupling contribution responsible for site energy modulation has been repackaged into a rescaling of the electronic coupling. This prescription is valid in the scenario where site energy fluctuations are primarily due to high-frequency intramolecular vibrations, rather than polarisation effects, and the timescale for relaxation of intramolecular phonons is much faster than the timescale for electronic motion, requiring $\omega_H \gg J_{max}$, where J_{max} is the largest electronic coupling present. Rubrene represents a borderline case, where we have $\omega_H/J_{max} \approx 1.4$, however it has been argued that the essential physics is still captured in this parameter regime.[30]

Mobility from Transient Localisation Theory

Table S1: TLT mobility (μ_a, μ_b) and % increase in mobility compared to the unstrained system using all three TLT approaches described previously. The error bars are equal to half the difference between the TLT mobility calculated using the electronic coupling distributions from one set of dimers ($J_{a,1}$, $J_{b,1}$) and the other ($J_{a,2}$, $J_{b,2}$).

Strain	Approach	μ_a / $cm^2(Vs)^{-1}$	μ_b / $cm^2(Vs)^{-1}$	% increase μ_a / μ_b
Unstrained	Literature	24.4[d], 15.3[e], 13.9[f], 20[g]	9.5[d], 1.26[e]	-
	1[a]	40.2 ± 1.9	10.3 ± 0.5	-
	2[b]	6.6 ± 0.0	1.6 ± 0.0	-
	3[c]	33.3 ± 1.2	7.4 ± 0.2	-
-0.8 % along $\vec{a}$	1[a]	44.1 ± 1.4	11.9 ± 0.7	9.8 / 16.3
	2[b]	7.0 ± 0.4	1.9 ± 0.2	6.2 / 13.4
	3[c]	35.6 ± 1.2	8.4 ± 0.6	7.0 / 13.7
-0.8 % along $\vec{b}$	1[a]	53.5 ± 0.0	13.8 ± 0.1	33.2 / 34.5
	2[b]	7.9 ± 0.0	2.0 ± 0.0	20.2 / 23.4
	3[c]	41.8 ± 0.3	9.3 ± 0.0	25.7 / 26.7

[a] No diagonal electron-phonon coupling, i.e., all site energies set to zero.

[b] Site energy fluctuations from Gaussian distribution corresponding to $\lambda = 0.152$ eV.

[c] Diagonal electron-phonon coupling accounted for via band renormalization.

[d] Ref. 19, computational study.

[e] Ref. 20, computational study.

[f] Ref. 31, experimental study.

[g] Ref. 32, experimental study.

References

(1) Kühne, T. D.; Iannuzzi, M.; Del Ben, M.; Rybkin, V. V.; Seewald, P.; Stein, F.; Laino, T.; Khaliullin, R. Z.; Schütt, O.; Schiffmann, F., et al. CP2K: An electronic structure and molecular dynamics software package-Quickstep: Efficient and accurate electronic structure calculations. *The Journal of Chemical Physics* **2020**, *152*, 194103.

(2) Lippert, B. G.; PARRINELLO, J. H.; MICHELE, A hybrid Gaussian and plane wave density functional scheme. *Molecular Physics* **1997**, *92*, 477–488.

(3) Lehtola, S.; Steigemann, C.; Oliveira, M. J.; Marques, M. A. Recent developments in libxc—A comprehensive library of functionals for density functional theory. *SoftwareX* **2018**, *7*, 1–5.

(4) Borštnik, U.; VandeVondele, J.; Weber, V.; Hutter, J. Sparse matrix multiplication: The distributed block-compressed sparse row library. *Parallel Computing* **2014**, *40*, 47–58.

(5) Marek, A.; Blum, V.; Johanni, R.; Havu, V.; Lang, B.; Auckenthaler, T.; Heinecke, A.; Bungartz, H.-J.; Lederer, H. The ELPA library: scalable parallel eigenvalue solutions for electronic structure theory and computational science. *Journal of Physics: Condensed Matter* **2014**, *26*, 213201.

(6) Marques, M. A.; Oliveira, M. J.; Burnus, T. Libxc: A library of exchange and correlation functionals for density functional theory. *Computer physics communications* **2012**, *183*, 2272–2281.

(7) Klimeš, J.; Bowler, D. R.; Michaelides, A. Chemical accuracy for the van der Waals density functional. *Journal of Physics: Condensed Matter* **2009**, *22*, 022201.

(8) Román-Pérez, G.; Soler, J. M. Efficient implementation of a van der Waals density

functional: application to double-wall carbon nanotubes. *Physical review letters* **2009**, *103*, 096102.

(9) VandeVondele, J.; Hutter, J. Gaussian basis sets for accurate calculations on molecular systems in gas and condensed phases. *The Journal of chemical physics* **2007**, *127*, 114105.

(10) Goedecker, S.; Teter, M.; Hutter, J. Separable dual-space Gaussian pseudopotentials. *Physical Review B* **1996**, *54*, 1703.

(11) Broyden, C. G. The convergence of a class of double-rank minimization algorithms 1. general considerations. *IMA Journal of Applied Mathematics* **1970**, *6*, 76–90.

(12) Monkhorst, H. J.; Pack, J. D. Special points for Brillouin-zone integrations. *Phys. Rev. B* **1976**, *13*, 5188–5192.

(13) VandeVondele, J.; Hutter, J. An efficient orbital transformation method for electronic structure calculations. *The Journal of chemical physics* **2003**, *118*, 4365–4369.

(14) Kolafa, J. Time-reversible always stable predictor–corrector method for molecular dynamics of polarizable molecules. *Journal of computational chemistry* **2004**, *25*, 335–342.

(15) Zhang, Y.; Manke, D. R.; Sharifzadeh, S.; Briseno, A. L.; Ramasubramaniam, A.; Koski, K. J. The elastic constants of rubrene determined by Brillouin scattering and density functional theory. *Applied Physics Letters* **2017**, *110*, 071903.

(16) Kondov, I.; Čížek, M.; Benesch, C.; Wang, H.; Thoss, M. Quantum Dynamics of Photoinduced Electron-Transfer Reactions in Dye- Semiconductor Systems: First-Principles Description and Application to Coumarin 343- TiO2. *The Journal of Physical Chemistry C* **2007**, *111*, 11970–11981.

(17) Futera, Z.; Blumberger, J. Electronic couplings for charge transfer across molecule/metal and molecule/semiconductor interfaces: Performance of the projector

operator-based diabatization approach. *The Journal of Physical Chemistry C* **2017**, *121*, 19677–19689.

(18) Bussi, G.; Donadio, D.; Parrinello, M. Canonical sampling through velocity rescaling. *The Journal of chemical physics* **2007**, *126*, 014101.

(19) Ruggiero, M. T.; Ciuchi, S.; Fratini, S.; D'avino, G. Electronic structure, electron-phonon coupling, and charge transport in crystalline rubrene under mechanical strain. *The Journal of Physical Chemistry C* **2019**, *123*, 15897–15907.

(20) Landi, A.; Peluso, A.; Troisi, A. Quantitative Prediction of the Electro-Mechanical Response in Organic Crystals. *Advanced Materials* **2021**, 2008049.

(21) Blumberger, J.; Sprik, M. Quantum versus classical electron transfer energy as reaction coordinate for the aqueous Ru 2+/Ru 3+ redox reaction. *Theoretical Chemistry Accounts* **2006**, *115*, 113–126.

(22) Ciuchi, S.; Fratini, S.; Mayou, D. Transient localization in crystalline organic semiconductors. *Physical Review B* **2011**, *83*, 081202.

(23) Fratini, S.; Mayou, D.; Ciuchi, S. The transient localization scenario for charge transport in crystalline organic materials. *Advanced Functional Materials* **2016**, *26*, 2292–2315.

(24) Nematiaram, T.; Ciuchi, S.; Xie, X.; Fratini, S.; Troisi, A. Practical computation of the charge mobility in molecular semiconductors using transient localization theory. *The Journal of Physical Chemistry C* **2019**, *123*, 6989–6997.

(25) Fratini, S.; Ciuchi, S.; Mayou, D.; De Laissardière, G. T.; Troisi, A. A map of high-mobility molecular semiconductors. *Nature materials* **2017**, *16*, 998–1002.

(26) Giannini, S.; Carof, A.; Ellis, M.; Yang, H.; Ziogos, O. G.; Ghosh, S.; Blumberger, J. Quantum localization and delocalization of charge carriers in organic semiconducting crystals. *Nature communications* **2019**, *10*, 1–12.

(27) Wang, J.; Wolf, R. M.; Caldwell, J. W.; Kollman, P. A.; Case, D. A. Development and testing of a general amber force field. *Journal of computational chemistry* **2004**, *25*, 1157–1174.

(28) Zhu, L.; Yi, Y.; Li, Y.; Kim, E.-G.; Coropceanu, V.; Bredas, J.-L. Prediction of remarkable ambipolar charge-transport characteristics in organic mixed-stack charge-transfer crystals. *Journal of the American Chemical Society* **2012**, *134*, 2340–2347.

(29) Holstein, T. Studies of polaron motion: Part II. The "small" polaron. *Annals of physics* **1959**, *8*, 343–389.

(30) Fetherolf, J. H.; Golež, D.; Berkelbach, T. C. A unification of the Holstein polaron and dynamic disorder pictures of charge transport in organic crystals. *Physical Review X* **2020**, *10*, 021062.

(31) Blülle, B.; Häusermann, R.; Batlogg, B. Approaching the trap-free limit in organic single-crystal field-effect transistors. *Physical Review Applied* **2014**, *1*, 034006.

(32) Podzorov, V.; Menard, E.; Borissov, A.; Kiryukhin, V.; Rogers, J. A.; Gershenson, M. Intrinsic charge transport on the surface of organic semiconductors. *Physical review letters* **2004**, *93*, 086602.
